# Vitamin C facilitates direct cardiac reprogramming by inhibiting reactive oxygen species

**DOI:** 10.1186/s13287-023-03615-x

**Published:** 2024-01-17

**Authors:** Juntao Fang, Qiangbing Yang, Renée G. C. Maas, Michele Buono, Bram Meijlink, Dyonne Lotgerink Bruinenberg, Ernest Diez Benavente, Michal Mokry, Alain van Mil, Li Qian, Marie-José Goumans, Raymond Schiffelers, Zhiyong Lei, Joost P. G. Sluijter

**Affiliations:** 1 Experimental Cardiology laboratory, Department of Cardiology, University Medical Center Utrecht, Utrecht, The Netherlands; 2 CDL Research, University Medical Center Utrecht, Utrecht, The Netherlands; 3 Circulatory Health Laboratory, UMC Utrecht, Regenerative Medicine Center Utrecht, University Utrecht, 3508 GA Utrecht, The Netherlands; 4 McAllister Heart Institute, University of North Carolina, Chapel Hill, NC USA; 5 Department of Cell and Chemical Biology, Leiden University Medical Centre, Leiden, The Netherlands

**Keywords:** Vitamin C, Cardiac reprogramming, ROS, Cardiac regeneration

## Abstract

**Background:**

After myocardial infarction, the lost myocardium is replaced by fibrotic tissue, eventually progressively leading to myocardial dysfunction. Direct reprogramming of fibroblasts into cardiomyocytes via the forced overexpression of cardiac transcription factors Gata4, Mef2c, and Tbx5 (GMT) offers a promising strategy for cardiac repair. The limited reprogramming efficiency of this approach, however, remains a significant challenge.

**Methods:**

We screened seven factors capable of improving direct cardiac reprogramming of both mice and human fibroblasts by evaluating small molecules known to be involved in cardiomyocyte differentiation or promoting human-induced pluripotent stem cell reprogramming.

**Results:**

We found that vitamin C (VitC) significantly increased cardiac reprogramming efficiency when added to GMT-overexpressing fibroblasts from human and mice in 2D and 3D model. We observed a significant increase in reactive oxygen species (ROS) generation in human and mice fibroblasts upon Doxy induction, and ROS generation was subsequently reduced upon VitC treatment, associated with increased reprogramming efficiency. However, upon treatment with dehydroascorbic acid, a structural analog of VitC but lacking antioxidant properties, no difference in reprogramming efficiency was observed, suggesting that the effect of VitC in enhancing cardiac reprogramming is partly dependent of its antioxidant properties.

**Conclusions:**

Our findings demonstrate that VitC supplementation significantly enhances the efficiency of cardiac reprogramming, partially by suppressing ROS production in the presence of GMT.

**Graphical abstract:**

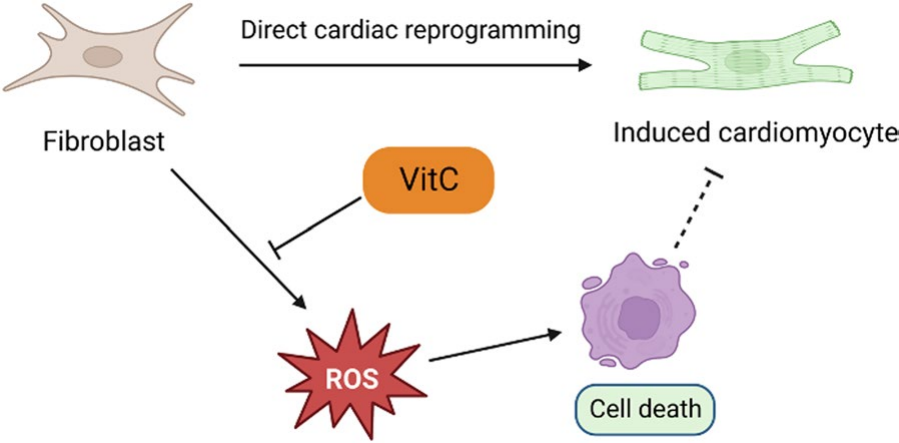

**Supplementary Information:**

The online version contains supplementary material available at 10.1186/s13287-023-03615-x.

## Background

Cardiovascular disease (CVD) remains a leading cause of death worldwide, with the associated costs expected to rise further [1]. Myocardial infarction (MI) is a primary contributor to CVD, in which lost myocardium is replaced with fibrotic tissue due to the limited regenerative capacity of cardiomyocytes. An increasing number of patients post-MI develop heart failure [2], a state in which the heart provides too little oxygenated blood into the circulation [3]. Currently, there are only a few curative treatments available for end-stage heart failure patients post-MI, limited to left ventricle assistant devices (LVADs) and heart transplantation [4]. However, LVADs can lead to complications including bleeding, infection, thrombosis, and dysrhythmia and typically only provide support for up to 5 years [5]. Heart transplantation serves as the ultimate solution for end-stage heart failure patients, but the application is limited due to donor scarcity and immune rejection issues [6]. Consequently, numerous studies have focused on repopulating the lost myocardial tissue post-MI in an attempt to regenerate the infarcted region and restore heart function at the basis of the problem and repopulate lost tissues. One such approach involves cell-based strategies that generate functional cardiomyocytes through cellular reprogramming, in which non-cardiomyocytes can be converted into cardiomyocytes via the overexpression of cardiac-specific transcription factors such as Gata4, Mef2c, and Tbx5 (GMT) [7]. Several studies have demonstrated that cardiac reprogramming can reduce scar size and improve cardiac function in mice post-MI [7–10]. This strategy offers several advantages, including the prevention of cell wash-out upon injection due to the presence of fibroblasts in the infarcted myocardial area, a reduction in fibrosis when fibroblasts can be converted into induced cardiomyocytes (iCMs), and the cell-free approach, thereby avoiding wash-out upon injection, a decreased likelihood of immune rejection and occurrence of arrhythmias. Unfortunately despite previous attempts, the direct cardiac reprogramming efficiency remains very low, particularly in human cells [11]. Therefore, further investigation of the underlying molecular mechanisms is required.

Various approaches have been explored to identify gene combinations or conditions that enhance cardiac reprogramming, such as altering GMT stoichiometry, adding supplementary growth factors like fibroblast growth factor (FGF) and vascular endothelial growth factor (VEGF), modulating different signaling pathways such as the Hippo pathway, or manipulating gene expression including Beclin1 and Bmi1 [8, 12–18]. However, most of these approaches and improvements have been studied and observed primarily in mouse embryonic fibroblasts (MEFs). In the current study, we investigated the impact of several factors on enhancing GMT-reprogramming efficiency in both MEFs and human fetal cardiac fibroblasts. We selected a panel of small molecules previously reported to facilitate cardiac differentiation or hiPSC reprogramming [19–25]. The ability of these molecules alone to sufficiently enhance cardiac reprogramming efficiency, however, remains uncertain.

Here, we present the first evidence that Vitamin C (VitC) significantly promotes the direct reprogramming of fibroblasts by GMT into cardiomyocytes, partially mediated by reactive oxygen species (ROS) reduction. Our findings offer a cost-effective, safe, and efficient strategy for generating iCMs.

## Materials and methods

### Generation of inducible cardiac reprogramming reporter in mouse embryonic fibroblasts

Mouse embryonic fibroblasts (MEF) were obtained from the aMHC/GFP transgenic mouse on embryonic day 13.5, as previously described [7, 9, 26]. In short, hearts were digested with 0.1% trypsin and plated on plastic dishes. Attached fibroblasts were cultured for 7 days, and α-MHC-GFP^+^ cells were sorted and cultured in DMEM/M199 medium containing 10% FBS at a density of 10^4^/cm^2^.

To generate a MEF GMT cell line, MEFs were transfected with a PiggyBac vector [27] carrying an inducible TetOn3G promotor, the mouse GMT reprogramming factors, and mCherry. After co-transfection by using Lipofectamine 3000 (Invitrogen, Catalog number: L3000001), the cells were selected with puromycin at a final concentration of 2 µg/ml for 3 days (Gibco, A11138-03) to obtain a stable cell line.

### Generation of inducible cardiac reprogramming human fetal cardiac fibroblast (hFCFs) cell line

Human fetal heart tissue was obtained by individual permission using standard written informed consent procedures and prior approval of the ethics committee of the Leiden University Medical Center, the Netherlands (No. METC P08-087). This is in accordance with the principles outlined in the Declaration of Helsinki for the use of human tissue or subjects. Human fetal cardiac fibroblasts (hFCFs) cell line carrying an inducible cardiac reprogramming cassette was created with the PiggyBac (PB) transposon system as described above. hFCFs were transfected with either a GMT vector or an empty vector (Neg) together with a transposase plasmid. As a control, the Neg hFCFs cell line was created containing the same TetOn 3G promotor and mCherry tag, but not containing the reprogramming cassette. After co-transfection by using Lipofectamine 3000 (Invitrogen, Catalog number: L3000001), the cells were selected with puromycin at a final concentration of 2 µg/ml (Gibco, A11138-03) to obtain a stable cell line. Due to the tetOn3G promotor, it is possible to activate and inactivate the reprogramming cassette at every preferred time point by adding or removing doxycycline (Doxy) [26].

### Cardiac reprogramming induction

Cells were seeded at a density of 5 × 10^4^ cells per well in a 6-well plate coated with 0.1% gelatin and cultured in the reprogramming medium (DMEM with 10% FBS and 1% P/S). Reprogramming medium was changed every other day. Doxy (1 μg/ml, Clontech, 631,311) was added one day after the cells were plated to start reprogramming and maintained during cardiac reprogramming. VitC (Sigma, A4544) was given with a final concentration of 20 µg/ml or at indicated concentrations. The day of Doxy administration was considered as day 0.

### Reactive oxygen species (ROS) activity detection

ROS was determined using a 2’,7’–dichlorofluorescin diacetate (DCFDA, ab113851) assay following manufacturer’s instruction. In brief, cells were washed and trypsinized into single cells, and then cells were stained with DCFDA for 30 min (without washing) at 37 ℃ and analyzed by Cytoflex (Beckman Coulter, #A00-1-1102). Data were analyzed using FlowJo 10.4.

### qRT-PCR

Total RNA was isolated at the indicated time points using the Nucleospin RNA isolation kit from Macherey–Nagel (740.955.250). The qScript cDNA synthesis kit from Quanta BioSciences (95,047–100) was used to synthesize cDNA from the isolated RNA samples (300 ng per sample). The quantitative real-time polymerase chain reaction (qRT-PCR) was performed with iQ SYBR Green Supermix (Bio-Rad). *β-actin* was selected as the housekeeping gene and was used for the calculation of normalized gene expression levels (ΔCt). The primers required in this study are listed in Table 1 and purchased from Integrated DNA Technologies.

**Table 1:** Primers list used for qPCR experiments

Genes	Forward primer	Reverse primer
*Human genes*		
GATA4	CGACACCCCAATCTCGATATG	GTTGCACAGATAGTGACCCGT
MEF2C	CCAACTTCGAGATGCCAGTCT	GTCGATGTGTTACACCAGGAG
TBX5	CTGTGGCTAAAATTCCACGAAGT	GTGATCGTCGGCAGGTACAAT
ACTN2	CAAACCTGACCGGGGAAAAAT	CTGAATAGCAAAGCGAAGGATGA
MYH6	GCCCTTTGACATTCGCACTG	GGTTTCAGCAATGACCTTGCC
P53	GAGGTTGGCTCTGACTGTACC	TCCGTCCCAGTAGATTACCAC
β-Actin	GATCGGCGGCTCCATCCTG	GACTCGTCATACTCCTGCTTGC
*Mouse genes*		
*Gata4*	TCAACCGGCCCCTCATTAAG	GTGGTGGTAGTCTGGCAGT
*Mef2c*	ATCCCGATGCAGACGATTCAG	AACAGCACACAATCTTTGCCT
*Tbx5*	GGCATGGAAGGAATCAAGGTG	TTTGGGATTAAGGCCAGTCAC
*P53*	CTCTCCCCCGCAAAAGAAAAA	CGGAACATCTCGAAGCGTTTA
β-Actin	GTGACGTTGACATCCGTAAAGA	GCCGGACTCATCGTACTCC

### NGS RNA sequencing

RNA concentration and integrity were analyzed with the Agilent 2100 Bio analyzer prior to proceeding with sequencing (Agilent RNA 6000 Nano Kit). RNA concentration for each single sample must be at least more than 15 ng/ul, and the total RNA amount is no less than 0.3ug. The library construction kit is MGIEasy RNA Directional Library Prep Set (1,000,006,385), made by MGI. Sequencing was performed on the DNBSEQ-G400 platform with PE150. The sequencing data were filtered with SOAPnuke by (1) removing reads containing sequencing adapter; (2) removing reads whose low-quality base ratio (base quality less than or equal to 15) is more than 20%; (3) removing reads whose unknown base (‘N’ base) ratio is more than 5%, afterwards clean reads were obtained and stored in FASTQ format. The clean reads were mapped to the reference genome using HISAT2. After that, Ericscript (v0.5.5) and rMATS (V3.2.5) were used to detect fusion genes and differential splicing genes (DSGs), respectively. Bowtie2 was applied to align the clean reads to the gene set, a database built by BGI (Beijing Genomic Institute in ShenZhen), in which known and novel, coding and noncoding transcripts were included. Expression level of each gene was calculated by RSEM (v1.3.1). Read counts obtained from RNA sequencing data were normalized, and differential gene expression between groups was performed using DESeq2(v1.4.5) in R. The screening criteria for DEGs were *P*-value < 10^–6^ and Log2 fold change ≥ 0.5 and ≤ (− 0.5). Pathway enrichment analysis was performed using differentially upregulated genes, and the package “EnrichR” enriched for “GO_Biological_Process_2015” was used. Gene expression profiles were also analyzed using Omics Explorer 3.2 (Qlucore), and principal component analysis (PCA) was used to visualize the data. Gene set enrichment analysis (GSEA) was also performed with Qlucore to determine whether a gene set of interest was significantly enriched in one condition compared to another. Gene sets with (false discovery rate [FDR] < 0.1) were considered significantly enriched in the comparison made. RNA sequencing and data mapping are conducted in BGI Hongkong Tech Solution NGS Lab.

### Fluorescence-activated cell sorting (FACS)

For MEFs, cells from different groups were first washed with PBS, detached by trypsin and collected in 15-ml Eppendorf tube, filtered with Flowmi™ Cell Strainers, 40 um (southern labware, BAH136800040) and were analyzed by Cytoflex flow cytometer (Beckman Coulter, #A00-1-1102). For hFCFs, cells were washed and fixated in 70% cold ethanol for 30 min. Later, cells were washed twice with PBS and blocked with 5% goat serum for 30 min, washed twice with PBS and stained with primary antibody α-Actinin (Sigma, A7811, 22ug/ml) overnight at 4 ℃, washed with PBS twice and stained with goat anti-mouse IgG Alexa Fluor 488 s antibody (Invitrogen, A11001, in 1:400 dilution) for one hour at room temperature, and then washed with PBS twice, filtered with Flowmi™ Cell Strainers, 40 um and analyzed by Cytoflex flow cytometer in the similar setting.

### Spheroid generation

For MEFs spheroid formation, 0.5 million cells were added to one well of a 6-well ultra-low attachment well plate (Corning) in DMEM + 10% FBS + 1% PS with 10% KOSR and 10 μM Y-27632. Spheroid plates were kept on a rotation plate, 70 rpm at 37 °C and 5% CO^2^. Medium change was performed after 24 h with the basal medium without KOSR and Y-27632. After 3 days, the spheroids were treated with Doxy at a final concentration of 1 µg/ml with/without vitamin C at a final concentration of 20 µg/ml. After 7 days, the spheroids were collected and used for flow cytometry and immunofluorescent analysis.

### Spheroid immunolabeling and confocal imaging

For immunofluorescence staining, an optimized protocol was used that allows imaging with single-cell resolution of the whole 3D microtissues [28]. In short, spheroids were collected by carefully aspirating media and adding 1 mL cold phosphate-buffered saline (PBS) with bovine serum albumin (BSA) (Roche)-coated tips into BSA-coated tubes. The spheroids were washed with 10 mL ice-cold PBS and spun down for 3 min at 70 × g and 4 °C. Next, the spheroids were resuspended in 1 mL 4% paraformaldehyde (PFA, Santa Cruz) solution and fixed for 45 min at 4 °C. After the fixation, the spheroids were spun down and washed with 10 mL ice-cold PBS for 3 times. Spheroids were then blocked with the spheroid washing buffer (SWB) consisting of 0.1% Triton X-100 (Sigma), 0.2% of 10% (w/v) SDS (Sigma) and 0.2% bovine serum albumin (BSA, Sigma) in PBS (store at 4 °C, up to 2 weeks) for 15 min at 4 °C. After fixation and blocking, the spheroids were transferred to a 24-well suspension plate where immunolabeling was performed. Spheroids were incubated with antibodies anti-GFP (goat, R1091P, Origene) to stain newly developed cardiomyocytes. DNA Hoechst stain for nuclei labeling (Thermo Fisher Scientific) was added together with the secondary antibodies (Donkey anti goat-488, Thermo Fisher Scientific, 0.5ug/ml). Specimens were mounted using FUnGI consisting of 50% (v/v) glycerol (Sigma), 9.4% (v/v) dH2O, 10.6 mM tris base (Roche), 1.1 mM EDTA (Sigma), 2.5 M fructose (Sigma) and 2.5 M urea (Sigma). Fluorescent imaging was performed on a SP8 Confocal Microscope (Leica). Optical sectioning along the Z axis was performed, and the images were projected into a single focal plane using the manufacturer’s software.

### Statistical analysis

Data were analyzed with GraphPad Prism 9, and comparisons were performed with a t-test (non-parametric tests) and a two-way ANOVA. Data were presented as mean ± SEM. A value of *p* < 0.05 is considered as significantly different.

## Results

### Establish and verification of the Doxy-inducible cardiac reprogramming model

**Fig. 1:**
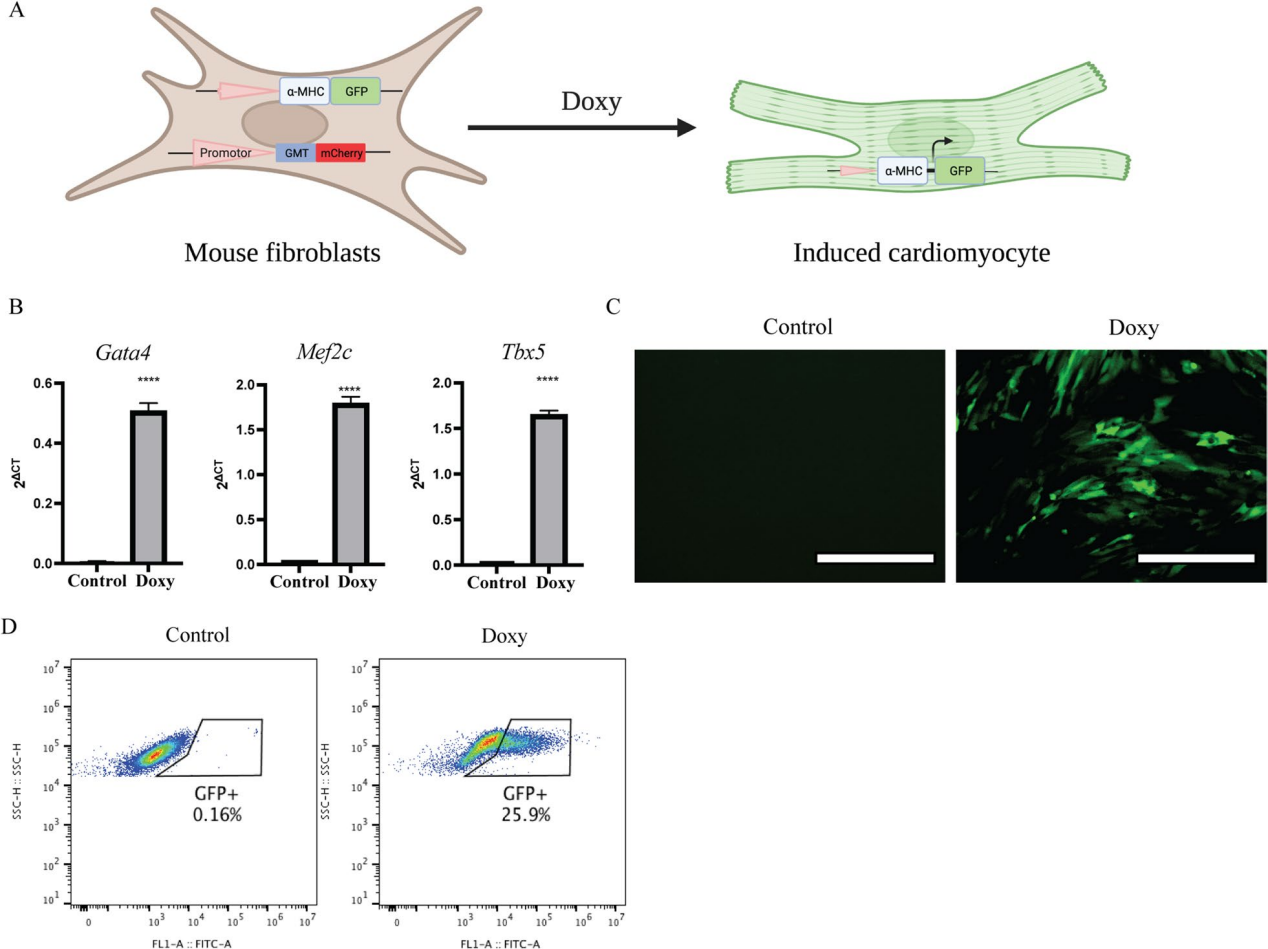
Forced expression of *Gata4*, *Mef2c* and *Tbx5* converts mouse embryonic fibroblasts (MEFs) into iCMs. **A** Strategy for creating a direct cardiac reprogramming system with MEFs containing an αMHC-GFP^+^ reporter. MEFs carrying a stable Doxy-inducible TetO promotor, mouse GMT reprogramming factors and a mCherry tag, can be converted into induced cardiomyocytes (iCM) upon exposure to Doxy. **B** Relative mRNA expression of GMT during reprogramming in GMT-transduced MEFs. RNA samples were collected from three independent experiments. **C** Representative fluorescent images of GFP expression in MEFs after Doxy exposure. Scale bar 200 μm. **D**, Representative FACS images of GFP^+^ MEFs upon Doxy exposure. Upon exposure of Doxy, GFP expression is induced in MEFs while not being present in non-exposed MEFs. Data were analyzed with t-test. ****p ≤ 0.0001 vs control. (Doxy: Doxycycline)

**Fig. 2:**
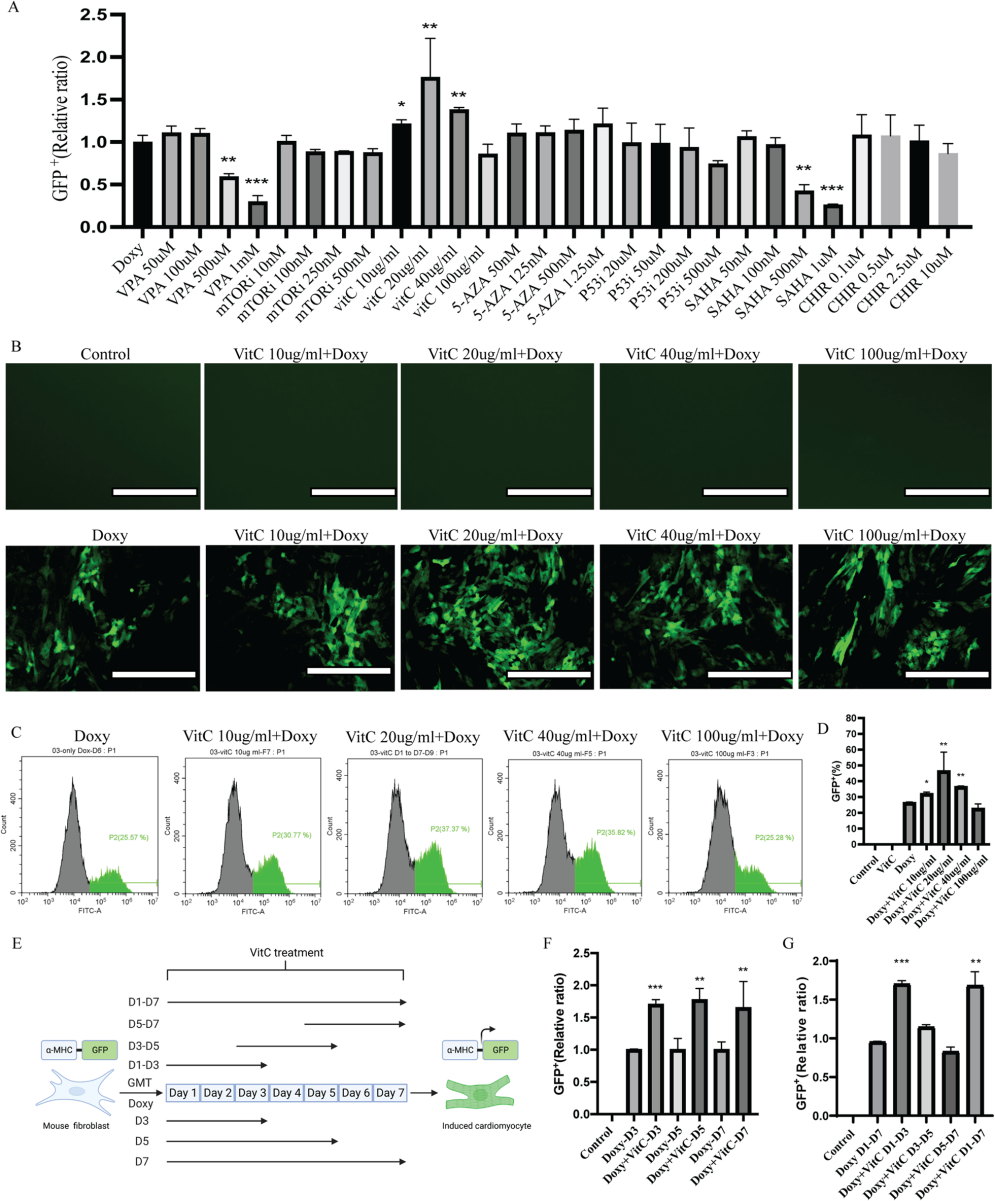
VitC promotes the direct reprogramming of MEFs into induced cardiomyocytes. **A** Summary of reprogramming efficiency with indicated small molecules to explore their cardiomyocyte forming-enhancing effect. Reprogramming efficiency of indicated conditions was measured by flow cytometric analyses. The GFP^+^ expression for each condition was normalized to the Doxy treated condition. **B** Representative fluorescence images of MEFs with or without Doxy exposure, together with exposure to different concentrations of VitC. Upon exposure to Doxy, cells started to express GFP while not being present in non-exposed MEFs, reflecting the success of cardiac reprogramming. Scale bar 200 μm. **C**, **D** Representative FACS images and statistical analysis of GFP^+^ MEFs with different concentrations of VitC treatment. **E** Experiment layout for exploring the duration effect of VitC on cardiac reprogramming. **F** Quantification of GFP^+^ MEFs of different duration with VitC administration. G, Quantification of GFP^+^ MEFs of different time slots with VitC administration. Mean values + SEM of three independent experiments is shown (*n* = 3). Data were analyzed with two-way ANOVA. *p ≤ 0.05 vs Doxy, **p ≤ 0.01 vs Doxy, ***p ≤ 0.001 vs Doxy. (Doxy: Doxycycline)

To facilitate our screening, we established a stable Doxy-inducible cardiac reprogramming cell model in which the Gata4, Mef2c and Tbx5 polycistron are under the control of a Doxy-inducible tetOn3G promoter (Figs. 1A and 2A). To validate the inducible cardiac reprogramming system, MEFs were treated with Doxy. Following Doxy induction, GMT gene expression inductions were confirmed in MEFs (Fig. 1B). After a successful cardiac reprogramming, GFP signal will become detectable in α-MHC-GFP MEFs by microscopic or flow cytometric analyses (Fig. 1C, D). Similarly, hFCFs were also treated with Doxy. After Doxy induction, mCherry expression was observed in hFCFs (Fig. 2B), and GMT gene expression inductions were confirmed in hFCFs as well (Fig. 2F). After a successful cardiac reprogramming, α-actinin-positive population will become detectable in hFCFs upon α-actinin staining with microscopic or flow cytometric analyses (Fig. 2C). Five days after Doxy addition, hFCFs cells with empty vectors (Neg) remained to display a cardiac fibroblast morphology with an elongated and stretched state (Fig. 2D). The morphology of hFCFs GMT cells, however, changed significantly and appeared like the previously reported cardiomyocyte progenitor cells (CMPCs), isolated from the human heart [30]. We measured cardiac-specific *MYH6* and *ACTN2* gene expression by qPCR and observed that the expression of these early cardiac-specific markers was significantly increased after Doxy induction, as compared to control groups (Fig. 2G). These results showed that the Doxy-inducible direct cardiac reprogramming system was functional, enabling both mice and human fibroblasts to be convert into induced cardiomyocytes (iCM), while the efficiency is still not very high.

### VitC promotes direct conversion of mouse embryonic fibroblasts into cardiomyocytes

**Fig. 3:**
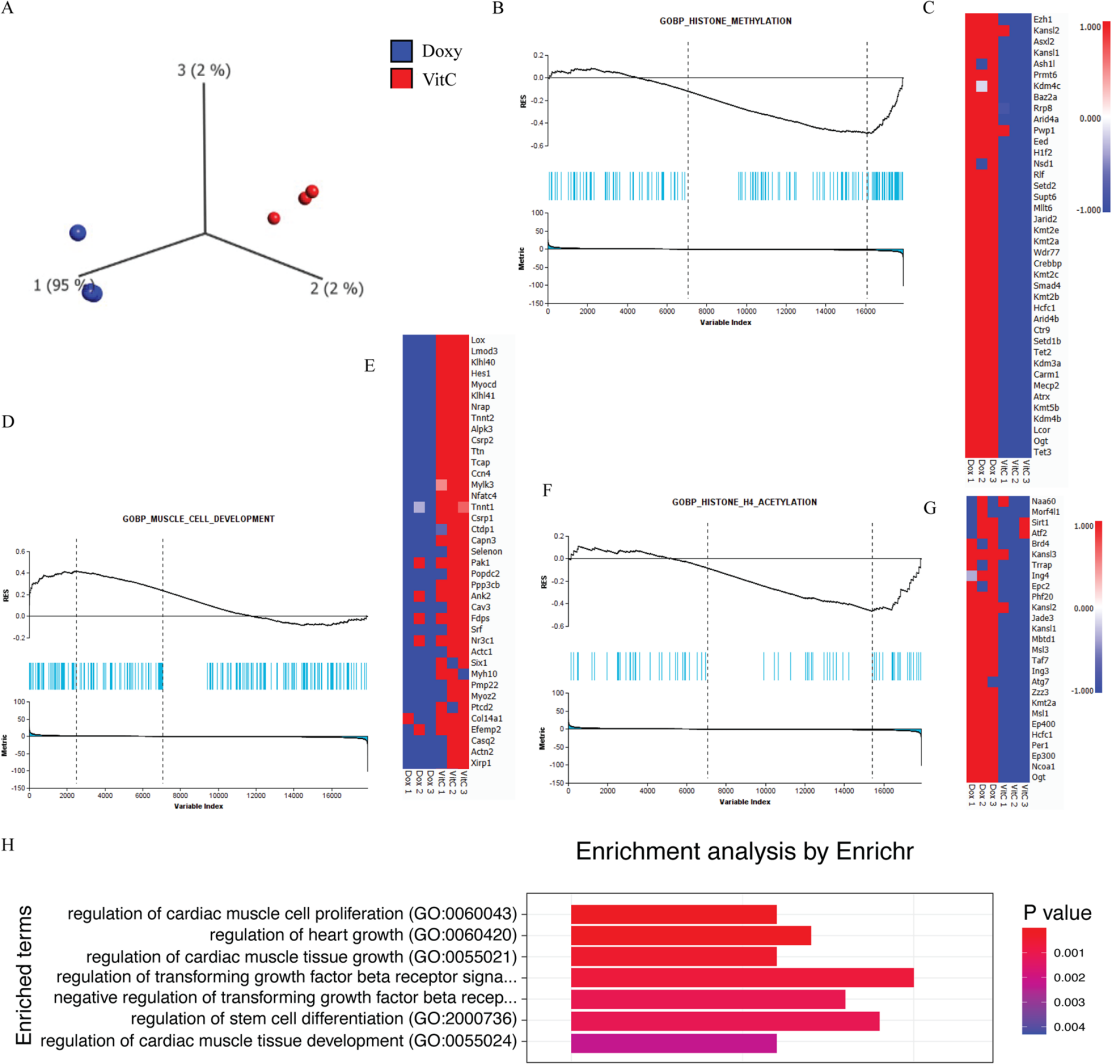
Analysis of differential expression upon GMT reprogramming and VitC exposure by RNA-Seq in MEFs. **A** PCA plot analysis was based on 622 differentially expressed genes between Doxy exposure alone and Doxy plus VitC (FDR = 0.1). The first principal components represent 95% of the variance in the RNA-seq data which indicated that the variance primarily comes from the difference between these two groups. **C**, **E**, **G**, heatmaps demonstrated that in muscle cell development pathway, genes are significantly upregulated in Doxy plus VitC group as compared with Doxy exposure alone, while in histone methylation and acetylation pathway, genes are significantly downregulated in Doxy plus VitC group (FDR = 0.1). **B**, **D** **F**, GSEA was performed to elucidate whether the applied gene set is statistically enriched in certain pathways, such as muscle cell development, histone methylation and acetylation pathway as indicated above. NES = 1.65, − 1.68 and − 1.42, respectively, FDR < 0.1. NES, normalized enrichment score. H, Enrichment analysis revealed several significant pathways in RNA-seq. Data were analyzed through Qlucore Omics Explorer and the package “EnrichR” enriched for “GO_Biological_Process_2015” was used. (Doxy: Doxycycline)

Many factors have been shown to potentially modulate cardiac reprogramming (Table 2), including histone deacetylase (HDAC) inhibitors Suberoylanilide hydroxamic acid (SAHA), and valproic acid (VPA), demethylating agent 5-azacytidine (5-AZA), Wnt agonists CHIR 99021, and ascorbic acid (VitC), mTOR and p53 inhibition [19, 22–25, 29]. We hypothesized that these molecules may enhance direct cardiac reprogramming of GMT. Therefore, we screened various concentrations of these molecules in the α-MHC-GFP/GMT MEF cell line (Fig. 3A). VPA (500 µM and 1 mM) and SAHA (500 nM and 1 µM), however, concentration dependently decreased the cardiac reprogramming efficiency. We assumed that this might be due to the toxicity of VPA and SAHA at high concentrations, whereas iCMs are more susceptible to this toxicity. Interestingly, we observed that VitC showed a consistent and robust cardiac reprogramming enhancing effect and achieved the highest proportion of GFP^+^ cells (Fig. 3A). In the absence of Doxy, no GFP signal was observed upon VitC exposure which indicates the robustness of our system (Fig. 3B top row). The GFP signal was clearly visible when treated for 7 days with Doxy and Doxy + VitC (Fig. 3B bottom row). These initial results were further validated by FACS analysis, demonstrating that approximately 25% GFP^+^ cells were observed 7 days after Doxy addition, whereas VitC (with a concentration of 10 µg/mL, 20 µg/mL and 40 µg/mL) can significantly increase the proportion of GFP^+^ cells to 32%, 46.4% and 36.4%, respectively, as compared to Doxy alone. The proportion of GFP^+^ cells decreased to 22.6% when treated with VitC at a concentration of 100 µg/mL (Fig. 3C, D), and this probably linked to increased cell death during reprogramming and exposure to VitC, as the iCMs are more sensitive to this response.

**Table 2:** Small molecules to enhance direct cardiac differentiation

Name	Characteristics	Refs.
VPA (Selleck, S3944)	Histone deacetylase (HDAC) inhibitor, enhanced induction of hiPSC	[22]
mTORi (Selleck, S1039)	Cell proliferation, survival and transcription regulation, enhanced induction of hiPSC	[25]
VitC (Sigma, A4544)	Antioxidant and cofactor for epigenetic modifiers, involved in cardiac differentiation from hiPSC	[19]
5-AZA (Selleck, S1782)	DNA methylation inhibitor, involved in cardiac differentiation from human embryonic stem cells	[29]
P53i (Selleck, S2929)	Transcription regulation, DNA damage and repair regulation, enhanced induction of hiPSC	[23]
SAHA (Selleck, S1047)	Histone deacetylase (HDAC) inhibitor, enhanced induction of hiPSC	[22]
CHIR-99021 (Selleck, S1263)	GSK-3 inhibitor, involved in cardiac differentiation from hiPSC	[24]

*VPA* Valproic acid, *mTORi* mammalian target of rapamycin inhibitor, *5-AZA* 5-azacytidine, *P53i* P53 inhibitor, *SAHA* suberoylanilide hydroxamic acid, CHIR-99021

To determine the exact phase of cardiac reprogramming in which VitC participates, we added VitC (20 μg/ml) in the early-phase (day 1–3), mid-phase (day 3–5), and late-phase (day 5–7) during reprogramming in the MEF cell line (Fig. 3E). Here, the addition of VitC increased the ratio of GFP^+^ cells again and upon exposure during all the phases (Fig. 3F). We treated MEFs with VitC (20 μg/ml) for the different durations (3 days, 5 days and 7 days) and observed that VitC promotes cardiac reprogramming at all the different time durations (Fig. 3F). Interestingly, we found that administration in none of these phases achieved the maximal effect observed upon exposure to VitC for the entire follow-up period of the cardiac reprogramming (Fig. 3G). These results suggested that the treatment of VitC is essential throughout the entire reprogramming process.

**Fig. 4:**
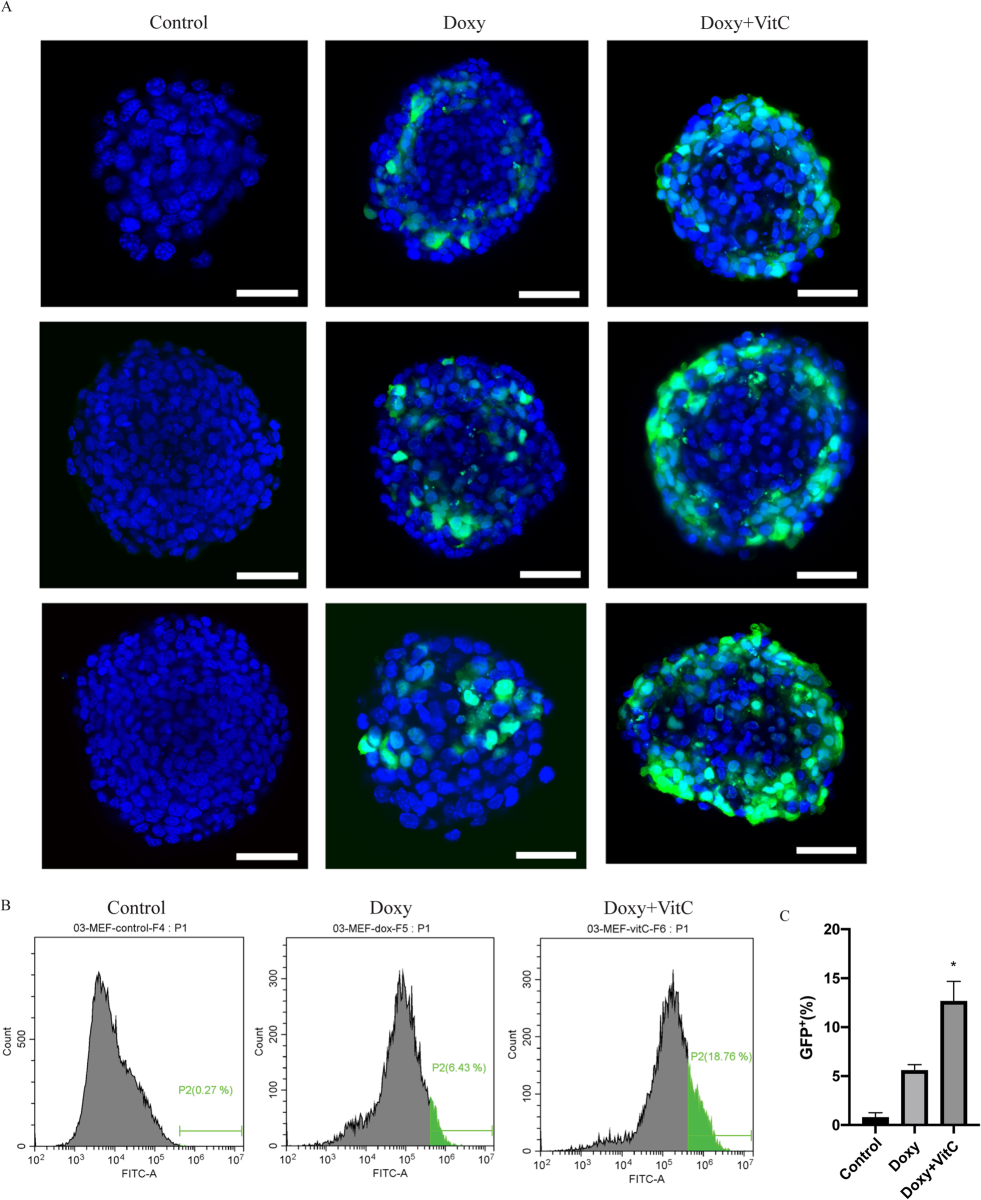
VitC promotes direct cardiac reprogramming efficiency of 3D spheroid derived from MEFs. **A** Immunofluorescence images of spheroid derived from MEFs, stained with GFP antibody. The blue color indicates DAPI staining and the green color indicates GFP staining that represented α-MHC signal. Upon exposure to Doxy, cells started to express GFP while not being present in non-exposed MEFs, reflecting the success of cardiac reprogramming. Scale bar 40 μm. **B**, **C**, Representative FACS image and quantification of GFP^+^ MEFs dissociated from MEFs spheroid. Mean values + SEM of three independent experiments is shown (*n* = 3). Data were analyzed with two-way ANOVA. *p ≤ 0.05 vs Doxy. (Doxy: Doxycycline)

To further confirm the robust cardiac inducing effect of VitC on direct cardiac reprogramming efficiency and explore the underlying mechanisms, we performed RNA sequencing to identify the differentially expressed genes between Doxy alone and Doxy plus VitC group. In total, 622 genes were found to be differentially expressed between these two groups. Transcriptional differences within these two groups were visualized in a PCA plot (Fig. 4A). To investigate the transcriptional differences between these two groups further, differential gene expression analysis was performed. Heatmap shows that gene sets related to cardiac muscle cell development were significantly upregulated in Doxy plus VitC group in the comparison of the control group and certain cardiac genes such as *TNNT2*, *ACTC1* and *ACTN2* were demonstrated to be significantly increased in VitC treated group, as compared to Doxy alone (Fig. 4E). We performed GSEA to identify statistically enriched gene sets in transcriptomic data between these two groups and found that VitC treatment displayed a significant enrichment of genes associated with muscle cell development during cardiac reprogramming (Fig. 4D). Furthermore, we also observed that epigenetic modification changes were occurring upon VitC exposure, including histone methylation and histone acetylation (Fig. 4B, F). Heatmap analyses show that gene sets related to histone methylation and histone acetylation were significantly downregulated in Doxy plus VitC group (Fig. 4C, G). Enrichment analysis of differentially expressed genes revealed the presence of ongoing heart development processes (Fig. 4H).

### VitC facilitates cardiac reprogramming in 3D MEF spheroids

**Fig. 5:**
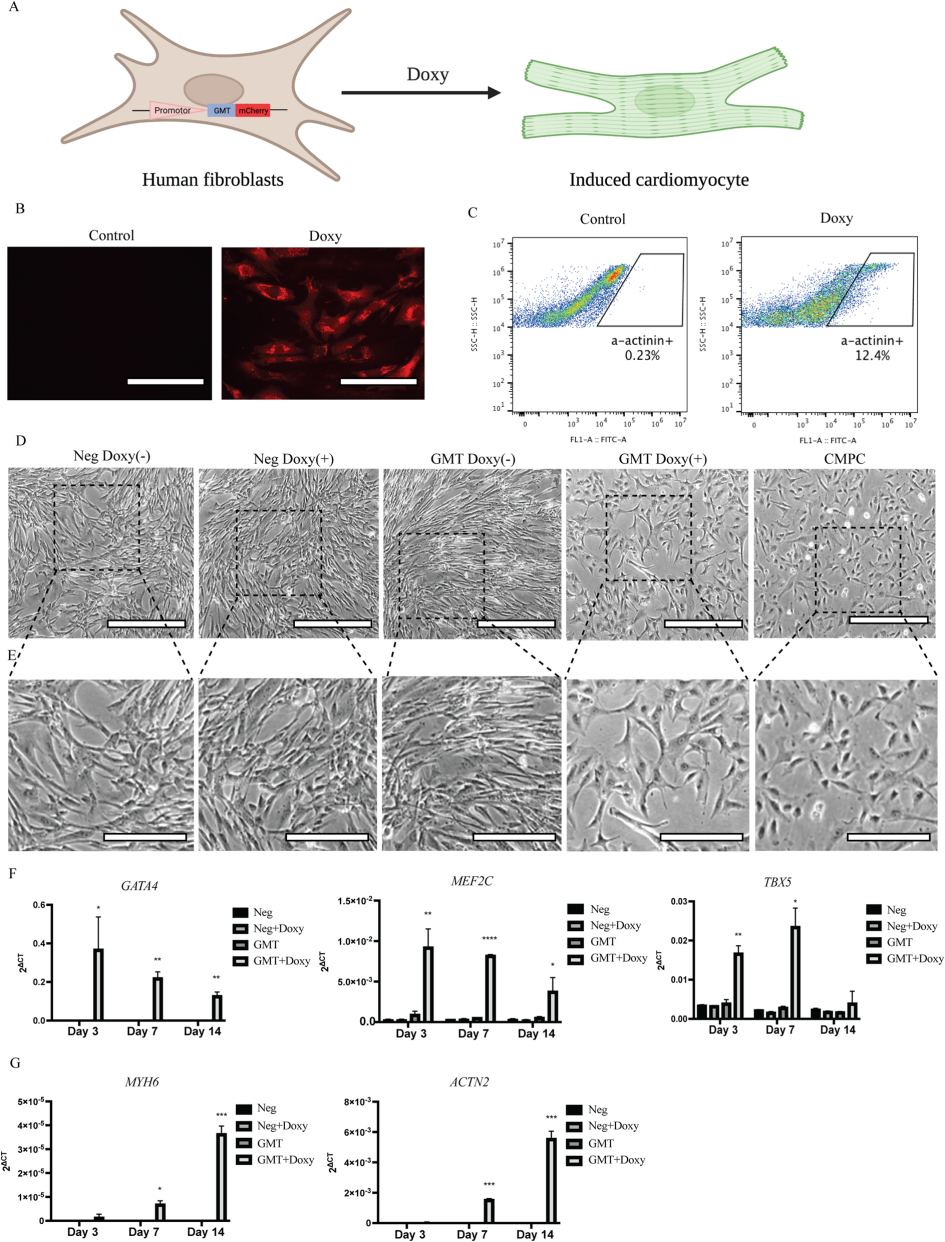
Forced expression of* GATA4*, *MEF2C* and *TBX5* converts human fetal cardiac fibroblast (hFCFs) into iCMs. **A** Strategy for creating a direct cardiac reprogramming system with hFCFs. hFCFs carrying a stable Doxy-inducible TetO promotor, mouse GMT reprogramming factors and a mCherry tag, can be converted into iCM upon exposure to Doxy. **B** Representative fluorescent images of mCherry expression in hFCFs after Doxy exposure. Upon exposure to Doxy, cells started to express mCherry while not being present in control cells, reflecting the presence of GMT factors. Scale bar 200 μm. **C** Representative FACS images of a-actinin staining positive hFCFs upon Doxy exposure. Upon exposure to Doxy, cardiac marker a-actinin expression is induced in hFCFs while not being present in non-exposed hFCFs. **D** Light microscopy images of GMT or Neg-transduced hFCFs on day 5, compared to cultured cardiomyocyte progenitor cells (CMPCs). Scale bar 500 μm. **E**, Magnified images from (**D**), Scale bar 250 μm. **F**, **G** Relative mRNA expression of GMT and cardiac genes *MYH6* and *ACTN2* during reprogramming in GMT or Neg-transduced hFCFs at different time points. RNA samples were collected from three independent experiments. Mean values + SEM of three independent experiments is shown (*n* = 3). Data were analyzed with two-way ANOVA. *p ≤ 0.05 vs GMT, **p ≤ 0.01 vs GMT, ***p ≤ 0.001 vs GMT and ****p ≤ 0.0001 vs GMT. (Doxy: Doxycycline; Neg: Empty vector)

To further explore if this VitC effect could be validated in a 3D environment, representing a more translational in vitro model [31, 32], MEF-containing spheroids were generated and treated with VitC for 7 consecutive days. Hereafter, the spheroids were either fixed by 4% PFA for GFP staining or dissociated into single cells for flow cytometry measurements. We observed that the expression of GFP was indeed increased in the VitC group compared to Doxy alone (Fig. 5A), which is consistent with the Flow cytometry results, where VitC addition significantly increases the GFP^+^ population from 5.5 to 11.2% (Fig. 5B, C). These results indicate that VitC is also sufficient to enhance the direct cardiac reprogramming efficiency in a 3D in vitro cell model.

### VitC enhances direct reprogramming from 2 and 3D cultured hFCFs into cardiomyocytes via suppression of ROS generation

**Fig. 6:**
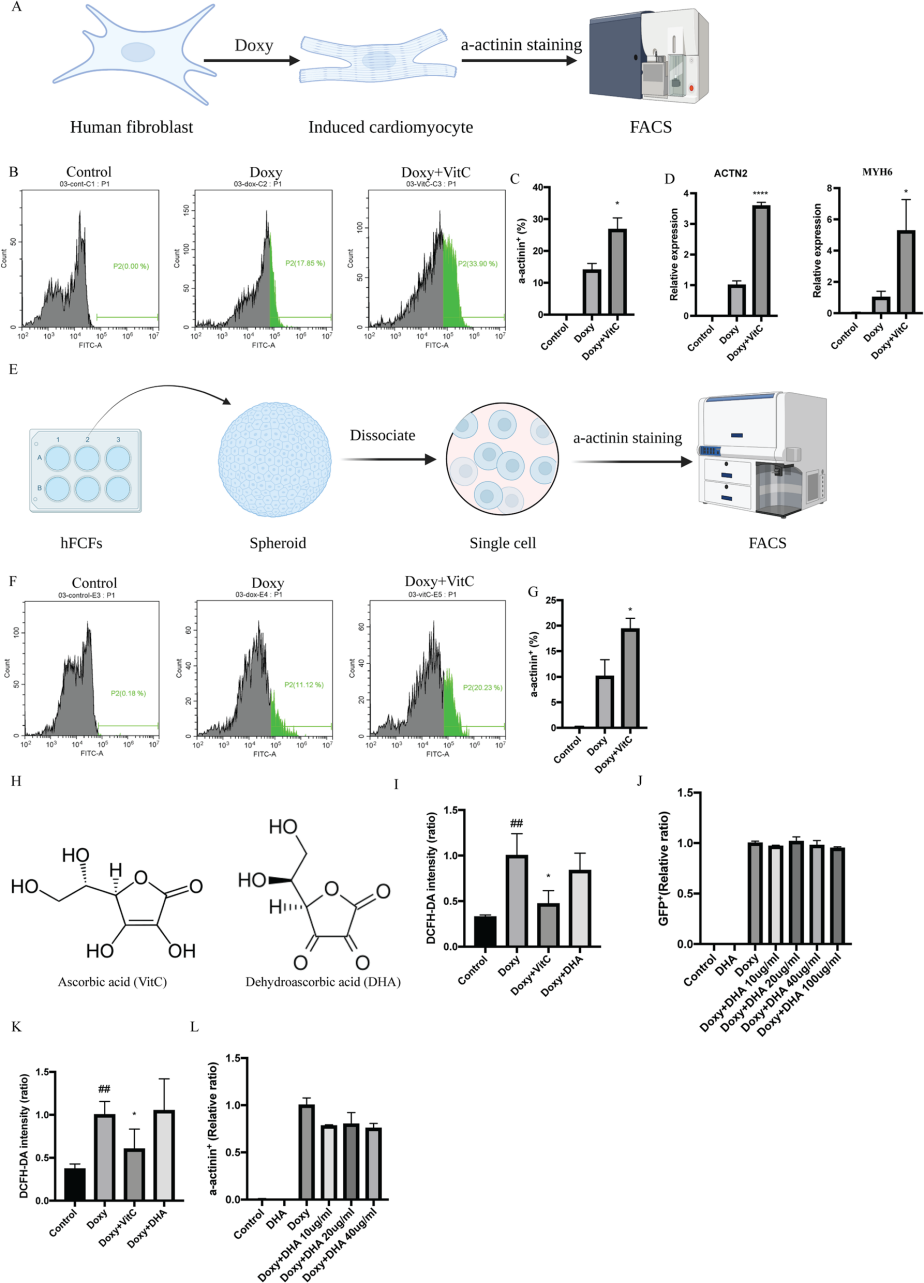
VitC enhances direct conversion of cardiomyocytes from hFCFs via ROS suppression. **A** Schematic overview of direct cardiac reprograming in hFCFs. **B** Representative FACS images of α-actinin^+^ hFCFs upon Doxy exposure. **C** Statistical analysis of α-actinin^+^ hFCFs upon Doxy exposure. **D** Relative mRNA expression of cardiac genes *ACTN2* and *MYH6* seven days after reprogramming. **E** Schematic overview of direct cardiac reprograming in spheroid derived from hFCFs. **F**, **G** Representative FACS images and statistical analysis of α-actinin^+^ spheroid derived from hFCFs. **H** Chemical structure of ascorbic acid and dehydroascorbic acid. **I** Flow cytometry analysis of DCFDA detection to measure MEFs ROS production upon Doxy exposure, treated with ascorbic acid or dehydroascorbic acid. **J** Quantification of GFP^+^ MEFs treated with ascorbic acid or dehydroascorbic acid. **K** Flow cytometry analysis of DCFDA detection to measure hFCFs ROS production upon Doxy exposure, treated with ascorbic acid or dehydroascorbic acid. **L** Quantification of α-actinin^+^ hFCFs upon Doxy exposure, treated with dehydroascorbic acid. Mean values + SEM of three independent experiments is shown (*n* = 3). Data were analyzed with two-way ANOVA. *p ≤ 0.05 vs Doxy, ****p ≤ 0.0001 vs Doxy, ^##^*p* ≤ 0.01 vs control. (Doxy: Doxycycline)

To determine if VitC exposure enhanced cardiac reprogramming efficiency in the human setting, we treated hFCFs with VitC with a concentration of 20 µg/ml for one week. hFCFs were collected either for α-actinin staining evaluated by FACS or for RNA isolation to determined mRNA expression of cardiac genes. We observed that VitC administration significantly increased the α-actinin positive cells, enhancing the percentage from 13.5 to 25.5%, as detected by FACS (Fig. 6B, C). Furthermore, mRNA expression level of cardiac genes *ACTN2* and *MYH6* (Fig. 6D) was increased 3.6- and 5.3-fold, respectively. Finally, in line with the 3D MEF spheroid results, we also observed that VitC significantly enhanced direct cardiac reprogramming efficiency of hFCFs spheroids (Fig. 6F, G), increasing the proportion of α-actinin^+^ positive cells from 13.9 to 26.7%.

ROS is an important player in hiPSCs reprogramming, having an inhibitory role, whereas VitC is known for its antioxidant properties [33]. To investigate if VitC enhances cardiac reprogramming via a ROS mediated mechanism, we determined ROS production by measuring DHA level upon Doxy-induced direct cardiac reprogramming. We observed a significant increase in ROS generation in MEFs and hFCFs upon Doxy induction, as compared to the control cell lines (Fig. 6I, K). ROS generation was subsequently reduced upon VitC treatment, associated with increased reprogramming efficiency (Figs. 4D, 6C, I, K). Interestingly, upon treatment with dehydroascorbic acid, a structural analog of VitC but lacking antioxidant properties (chemical structure shown in Fig. 6H), no difference in reprogramming efficiency was observed (Fig. 6I, L), suggesting that the effect of VitC in enhancing cardiac reprogramming is partly dependent of its antioxidant properties. We also tested the effect of α-lipoic acid (LA), an antioxidant, in MEFs with different concentration to see if it can boost cardiac reprogramming efficiency, and we observed LA with a concentration of 0.5 mM can significantly increase reprogramming efficiency (shown in Additional file 2; Figure S1), this further indicated that the reduction of ROS facilitates cardiac reprogramming efficiency. In addition, we found that direct cardiac reprogramming induces P53 expression while this was reduced with VitC treatment (shown in Additional file 2; Figure S2), indicating that VitC promotes cardiac reprogramming by reducing ROS-induced cell death.

## Discussion

Direct reprogramming of fibroblasts into induced cardiomyocytes (iCMs) offers a promising therapeutic approach for myocardial infarction (MI) patients by simultaneously eliminating fibroblasts and generating new cardiomyocytes in the infarcted region. However, the efficiency of direct cardiac reprogramming remains suboptimal. In this study, we investigated the effects of seven small molecules, on the efficiency of direct cardiac reprogramming in mouse and human fibroblasts. These molecules were selected based on their previously reported roles in cardiac differentiation and human-induced pluripotent stem cell (hiPSC) reprogramming. For instance, suberoylanilide hydroxamic acid (SAHA), also known as vorinostat and valproic acid (VPA) are well-known histone deacetylase (HDAC) inhibitor that have been reported to significantly enhance hiPSC reprogramming [26]. In particular, VPA emerged as a more potent modulator in cellular reprogramming through relaxation of chromatin structure, making it more accessible to transcription factors and other regulatory proteins [26, 34]; 5-azacytidine (5-AZA), a demethylating agent, was shown to enhance the differentiation of human embryonic stem cells into cardiomyocytes by DNA methylation regulation [35]. CHIR 99021, a Wnt agonist, can also robustly enhance cardiomyocyte differentiation from hiPSCs [29]. VitC, known as ascorbic acid, has emerged as a promising small molecule that can remarkably promote cardiac differentiation of embryonic stem cells (ESC) and hiPSCs, and enhances the generation of mouse and human-induced pluripotent stem cells [24, 25, 36]. The cell-cycle regulator P53 functions as an important safeguard and was also shown as a critical barrier to reprogramming process, P53 deletion significantly promotes hiPSC reprogramming [28]. In addition, mTOR inhibitor was also reported to enhance the generation of hiPSC [30]. Eventually, we identified vitamin C (VitC) as a potent enhancer of direct cardiac reprogramming efficiency. VitC, an essential nutrient for human health, has been widely utilized as a supportive compound in various cellular differentiation and reprogramming processes due to its beneficial properties, such as alleviating cell senescence and enhancing cellular proliferation [19, 21]. Our RNA sequencing data corroborate the transition of fibroblasts into induced cardiomyocytes, as evidenced by the upregulation of cardiac development genes such as *TNNT2*, *ACTC1* and *ACTN2* in the Doxy + VitC group. Interestingly, VitC-induced enhancement of cardiac reprogramming was also observed in a 3D spheroid model derived from both mouse embryonic fibroblasts (MEFs) and human fetal cardiac fibroblasts (hFCFs), indicating that VitC effectively promotes fibroblast-to-cardiomyocyte conversion in both the 2D and 3D environment. Intriguingly, other small molecules, such as 5-AZA, VPA, and SAHA, known to facilitate cardiac differentiation or hiPSC reprogramming, did not enhance direct cardiac reprogramming. Notably, higher concentrations of VPA and SAHA impeded cardiac reprogramming, while lower dosages exhibited no effect. This observation may be attributed to the toxicity of these compounds at higher concentrations.

ROS generation is elevated in the early stage of hiPSC reprogramming, and excessive ROS accumulation impairs hiPSC generation [37]. Molecules with antioxidant properties have been shown to boost the reprogramming of fibroblasts into hiPSCs such as resveratrol [35], N-acetylcysteine and VitC [38]. Nonetheless, the involvement of ROS in the process of direct cardiac reprogramming remains elusive. In our study, we discovered that VitC enhanced direct cardiac reprogramming efficacy and reduced ROS generation during the reprogramming process. In contrast, dehydroascorbic acid, which lacks potent antioxidant properties, did not affect cardiac reprogramming efficiency or ROS production. In this scenario, the function of VitC appears to be associated with its antioxidant activity. Further investigation into the underlying mechanisms is warranted.

The involvement of ROS in the process of direct cardiac reprogramming was unknown for a long time. Although vitamin E nicotinate, an antioxidant, has been shown to facilitate the application of direct cardiac reprogramming approach for cardiac repair [39], a direct link to the observed effects by the elimination of ROS was lacking. In our study, we demonstrated that the reduction of ROS by VitC facilitated cardiac reprogramming, this may bring new insights in direct cardiac conversion. Although this study suggests that enhanced direct cardiac reprogramming efficiency by VitC might be modulated by decreasing ROS production, it is possible that VitC effects are also mediated via e.g. enhanced epigenetic modifications. For instance, histone demethylases are important in regulating hiPSC reprogramming [34] and direct cardiac reprogramming [40], and VitC can affect the activity of these enzymes [41]; therefore, VitC might allow the direct reprogramming to proceed more smoothly by promoting histone demethylation. In our study, we observed that epigenetic modification changes were present in these processes, including histone methylation and histone acetylation, and thereby providing new insights into the mechanism of direct cardiac reprogramming. Despite our encouraging observation regarding VitC-mediated enhanced cardiac reprogramming, we did not see spontaneous beating of the cells in the end, even not with our iPS-derived cardiomyocyte culture media. We speculate that due to the reprogramming-induced cell death, cell lose essential interconnections and signal cascades. Besides, we merely have focused on in vitro models in our study. Further studies are required to investigate the ROS-mediated effect in vivo and explore new delivery systems to achieve higher efficiency for cardiac repair. In addition, exogenous ROS incubation, treatment of antioxidants in different induction timing, and loss-of-function studies of ROS-associated genes would be helpful to uncover the role of ROS in both in vitro and in vivo direct cardiac reprogramming in future studies [39].

Although VitC is known to boost induced pluripotent stem cells reprogramming and enhance cardiac differentiation of induced pluripotent stem cells [19, 21], its effect in facilitating somatic cell reprogramming into cardiomyocytes remained unclear. Our study provides the first evidence that VitC enables the enhancement of direct cardiac reprogramming efficiency of key cardiac transcriptional factors (GMT), either in human or mouse cells, as well as on both 2D and 3D level. Finally, we used RNAseq to get mechanistic insights and could demonstrated that ROS plays an important role during this process. The other identified mechanisms included epigenetic modifications, histone methylation, and histone acetylation, which could be additional potential mechanism that affect direct cardiac reprogramming efficiency, but will need more future investigations.

In conclusion, direct cardiac reprogramming represents a promising strategy for cardiac regeneration with the potential to become a novel therapeutic approach for heart failure. Our study, for the first time, demonstrates that VitC enhances the direct reprogramming of cardiomyocytes from fibroblasts using GMT transfection in vitro. This enhancement is partly attributed to a decrease in ROS production. Our findings pave the way for a combined gene and small molecule therapy for cardiac regeneration.

## Conclusions

Our findings demonstrate that VitC significantly enhances the efficiency of cardiac reprogramming, partially by suppressing ROS production in the presence of GMT, which provides novel insights into the regulation of direct cardiac reprogramming and its potential for therapeutic applications in cardiac repair.

## Supplementary Information

**Additional file 1**. Supplementary figure S1 and S2.

**Additional file 2**. Processed NGS data with and without VitC treatment.

## Data Availability

Our data have been deposited and can be fully accessed via: https://dataverse.nl/dataset.xhtml?persistentId=doi:10.34894/EZRRDE.

## Abbreviations

MI: Myocardial infarction
CVD: Cardiovascular disease
LVADs: Left ventricle assistant devices
FGF: Fibroblast growth factor
VEGF: Vascular endothelial growth factor
GMT: Gata4, Mef2c, Tbx5
hiPSC: Human-induced pluripotent stem cell
ESC: Embryonic stem cell
iCMs: Induced cardiomyocytes
MEFs: Mouse embryonic fibroblasts
hFCFs: Human fetal cardiac fibroblasts
CMPCs: Cardiomyocyte progenitor cells
VitC: Vitamin C
HDAC: Histone deacetylase
SAHA: Suberoylanilide hydroxamic acid
VPA: Valproic acid
5-AZA: 5-Azacytidine
mTORi: Mammalian target of rapamycin inhibitor
P53i: P53 inhibitor
DHA: Dehydroascorbic acid
ROS: Reactive oxygen species
PB: PiggyBac
Neg: Empty vector
Doxy: Doxycycline
DCFDA: 2’,7’-Dichlorofluorescin diacetate
RT-PCR: Real-time polymerase chain reaction
BSA: Bovine serum albumin
SWB: Spheroid washing buffer
PFA: Paraformaldehyde
ACTN2: Actinin alpha 2
MYH6: Myosin heavy chain 6
TNNT2: Troponin T2
ACTC1: Actin alpha cardiac muscle 1
