# Correction: Vitamin C facilitates direct cardiac reprogramming by inhibiting reactive oxygen species

**DOI:** 10.1186/s13287-024-04113-4

**Published:** 2024-12-24

**Authors:** Juntao Fang, Qiangbing Yang, Renée G. C. Maas, Michele Buono, Bram Meijlink, Dyonne Lotgerink Bruinenberg, Ernest Diez Benavente, Michal Mokry, Alain van Mil, Li Qian, Marie‑José Goumans, Raymond Schiffelers, Zhiyong Lei, Joost P. G. Sluijter

**Affiliations:** 1 Experimental Cardiology Laboratory, Department of Cardiology, University Medical Center Utrecht, Utrecht, The Netherlands; 2 CDL Research, University Medical Center Utrecht, Utrecht, The Netherlands; 3 Circulatory Health Laboratory, UMC Utrecht, Regenerative Medicine Center Utrecht, University Utrecht, 3508 GA Utrecht, The Netherlands; 4 McAllister Heart Institute, University of North Carolina, Chapel Hill, NC USA; 5 Department of Cell and Chemical Biology, Leiden University Medical Centre, Leiden, The Netherlands

**Correction: Stem Cell Research & Therapy (2024) 15:19** 10.1186/s13287-023-03615-x

The original article presents an error affecting Figure 2B.

In Fig. 2B, one of the images was inadvertently duplicated, resulting in an incorrect representation—there is no GFP/little signal in all Dox- groups, which is challenging to observe without an increase in figure intensity dramatically. This misrepresentation may impede the accurate interpretation of the data. The authors sincerely apologize for this oversight and wish to present the corrected Fig. 2B ahead to ensure the accuracy of the content.

**Fig. 2:**
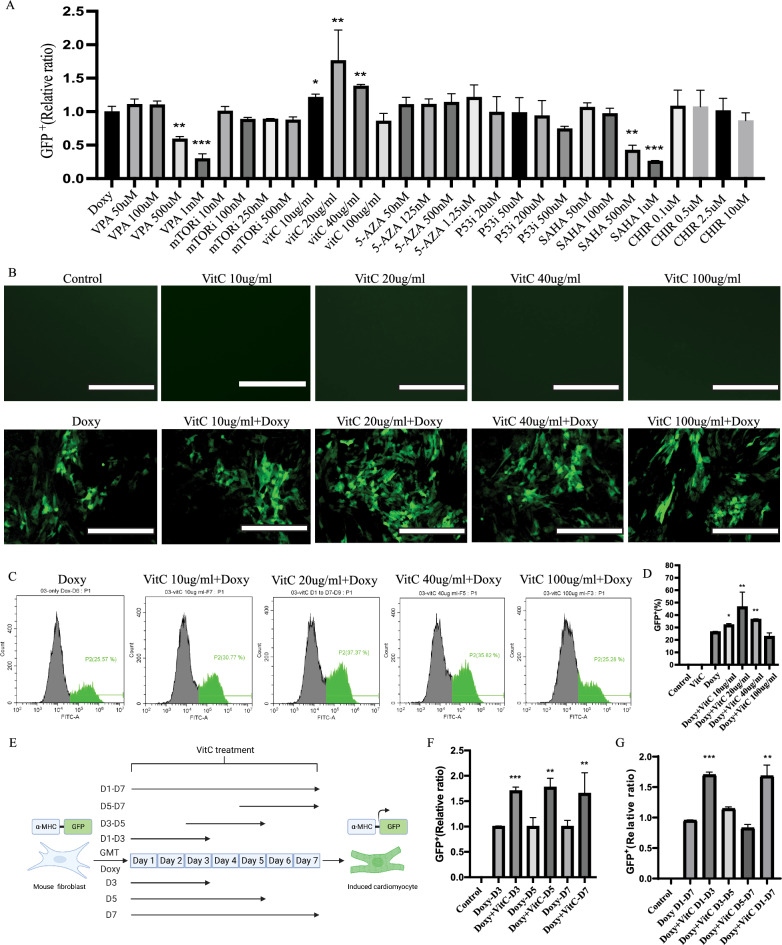
VitC promotes the direct reprogramming of MEFs into induced cardiomyocytes. **A** Summary of reprogramming efficiency with indicated small molecules to explore their cardiomyocyte forming-enhancing effect. Reprogramming efficiency of indicated conditions was measured by flow cytometric analyses. The GFP^+^ expression for each condition was normalized to the Doxy treated condition. **B** Representative fluorescence images of MEFs with or without Doxy exposure, together with exposure to different concentrations of VitC. Upon exposure to Doxy, cells started to express GFP while not being present in non-exposed MEFs, reflecting the success of cardiac reprogramming. Scale bar 200 μm. **C**, **D** Representative FACS images and statistical analysis of GFP^+^ MEFs with different concentrations of VitC treatment. **E** Experiment layout for exploring the duration effect of VitC on cardiac reprogramming. **F** Quantification of GFP^+^ MEFs of different duration with VitC administration. **G** Quantification of GFP^+^ MEFs of different time slots with VitC administration. Mean values + SEM of three independent experiments is shown (*n* = 3). Data were analyzed with two-way ANOVA. *p ≤ 0.05 vs. Doxy, **p ≤ 0.01 vs. Doxy, ***p ≤ 0.001 vs. Doxy. (Doxy: Doxycycline)

The conclusions of the original paper stands.

