# Supplementary material for: Vitamin C facilitates direct cardiac reprogramming by inhibiting reactive oxygen species
Source: Stem Cell Res Ther. 2024 Jan 17;15:19. doi: 10.1186/s13287-023-03615-x (PMC10792814; doi:10.1186/s13287-023-03615-x)
Supplement: Supplementary file 2 — Additional file 2. Processed NGS data with and without VitC treatment. [file 13287_2023_3615_MOESM2_ESM.docx]

# Supplementary Information

**Figure S1:** Reprogramming efficiency of α-lipoic acid (LA) with indicated concentrations were measured by flow cytometry. The GFP^+^ expression for each condition was normalized to the Doxy treated condition.

**

**Figure S2:** Relative mRNA expression of P53 seven days after reprogramming. *** p ≤ 0.001 vs control; # p ≤ 0.05 vs Doxy, ## p ≤ 0.01 vs Doxy.
